# A review of children with severe trauma admitted to pediatric intensive care in Queensland, Australia

**DOI:** 10.1371/journal.pone.0211530

**Published:** 2019-02-07

**Authors:** Mark G. Coulthard, Vanil Varghese, Lauren P. Harvey, Tona C. Gillen, Roy M. Kimble, Robert S. Ware

**Affiliations:** 1 Paediatric Intensive Care Unit, Queensland Children’s Hospital, Brisbane, Australia; 2 Academic Discipline of Paediatrics and Child Health, School of Clinical Medicine, The University of Queensland, Queensland Children’s Hospital, South Brisbane, Australia; 3 Paediatric Trauma Service, Queensland Children’s Hospital, Brisbane, Australia; 4 Centre for Children’s Burns and Trauma Research, Children’s Health Queensland, Brisbane, Australia; 5 Menzies Health Institute Queensland, Griffith University, Brisbane, Australia; UCLA, UNITED STATES

## Abstract

**Background:**

The aim of this study is to review patient characteristics, injury patterns, and outcomes of trauma cases admitted to pediatric intensive care in Children’s Health Queensland, Brisbane, Queensland, Australia.

**Methods:**

Routinely recorded data collected prospectively from the Children’s Health Queensland Trauma Service registry from November 2008 to October 2015 were reviewed. Demographic and clinical characteristics of trauma cases in children under 16 years of age are described, and their association with age and mortality analyzed.

**Results:**

There were 542 cases of pediatric trauma identified and 66.4% were male. The overall mortality since January 2012 was 11.1%. The median injury severity score (ISS) was 11 (IQR = 9–22), 48.2% (n = 261) had an ISS > 12 and 41.7% (n = 226) patients had an ISS > 15. The most common injury patterns were isolated head injury (29.7%; n = 161) and multiple trauma (31.2%; n = 169). In 28.4% of cases (n = 154) surgery was required. The home was reported to be the most common place of injury (37.6%; n = 204). Children aged 0–4 years were least likely to survive their injury (15.3% mortality) compared with the 5–9 (5.6% mortality) and 10–15 (9.0% mortality) age groups. Higher mortality was associated with more severe injuries, abdomen/spine/thorax injuries, inflicted injuries, drowning and hanging.

**Conclusion:**

This description of major pediatric trauma cases admitted to pediatric intensive care in Children’s Health Queensland, Australia, will inform future pediatric major trauma service requirements as it identifies injury patterns and profiles, injury severity, management and mortality across different age groups.

## Introduction

Trauma is a major public health problem worldwide across all age groups [1]. In the developed world, pediatric trauma is the leading cause of death and disability in children and adolescents [2, 3]. Trauma in an infant, child or adolescent creates special considerations with specific injury patterns, diagnosis and management due to the age-dependent anatomy, physiology and cognitive variability in children [4]. In the USA, it is estimated that one in four children will sustain an unintentional injury that requires medical care each year, and injury in children (aged 1–19 years) accounted for 34.4% of all childhood deaths in the USA in 2013 [5]. The costs of pediatric trauma include both the initial hospitalization and ongoing financial burden due to disability and future work losses [6]. Despite improvements in trauma care, a significant proportion of children live with long-term disability following major pediatric trauma [7].

The Australian Institute of Health and Welfare has published extensive data on the epidemiology and patterns of unintentional injury in infants and children [8]. Children admitted to a pediatric intensive care unit (PICU) have the highest morbidity and mortality due to the severity of their injuries [9]. Health outcomes of major trauma cases are improved when children are managed by a dedicated pediatric trauma service [10, 11]. Understanding patterns of pediatric injury is also crucial for developing and targeting effective prevention programs [12]. Further, there are reports of infants and children with major trauma admitted to pediatric intensive care units in other states and countries with integrated regionalized trauma systems [13–18]. To date there have been no published reports of infants and children admitted to pediatric intensive care due to major trauma in Queensland, Australia.

The aim of this study was to use trauma service registry data to report the demographic and clinical characteristics of children admitted to PICU in Brisbane with major trauma between 2008 and 2015. This would allow us to review our trauma service and could suggest areas for service improvement.

## Methods

### Setting

The state of Queensland, Australia, has a population of just over 4.5 million with 20% aged under 15 years. Approximately 50% of the population lives in the greater Brisbane metropolitan area, located in the south-east corner of the state. The remainder of the population is dispersed across an area of 1,727,000 square kilometres (twice the size of Texas) in several large regional metropolitan centres, numerous small towns, and rural and farming communities [19]. Prior to November 2014, the majority of major pediatric trauma cases were transferred to either the Mater Children’s Hospital, Brisbane or the Royal Children’s Hospital, Brisbane, and all burns patients were admitted to the Burns Centre at the Royal Children’s Hospital, Brisbane. Trauma processes from both children’s hospitals were similar, as the trauma surgery services were provided by paediatric surgeons who attended across both hospitals, and the emergency departments were under joint directorship from September 2010. The Royal Children’s Hospital and Mater Children’s Hospital, both tertiary children’s hospitals, merged into the new stand-alone quaternary pediatric service at the Lady Cilento Children’s Hospital, Brisbane, in November 2014. The Lady Cilento Children’s Hospital was renamed the Queensland Children’s Hospital (QCH) in 2018. The pediatric trauma service, located at QCH, is a level 1 trauma service, with a 24 hour per day trauma response team and the availability of a senior trauma surgeon. The QCH referral area covered by these tertiary pediatric centres and the level 1 pediatric trauma center at QCH includes northern New South Wales and > 80% of Queensland’s population (excluding the north and north-west of the state).

### Ethics

Ethics for this low and negligible risk project was approved by the Children’s Health Queensland Hospital and Health Service Ethics Committee (HREC Reference number: HREC/15/QRCH/203). The need for informed consent was waived. The data was accessed in an Excel spreadsheet and was deidentified before analysis and presentation.

### Data collection

Trauma data are prospectively collected by the Children’s Health Queensland Trauma Service registry. The trauma service registry records demographic and clinical characteristics of all major pediatric trauma cases admitted to QCH including all transfers and retrievals from Queensland and northern New South Wales. We investigated data from the Children’s Health Queensland Trauma Service registry that were prospectively collected between November 2008 and October 2015 for children aged < 15 years admitted to the PICUs at the Royal Children’s Hospital and Mater Children’s Hospital, and from November 2014, the QCH. The Children’s Health Queensland Trauma Service registry is maintained by a data manager at the QCH, who is responsible for the development, coordination and maintenance of the database. The data manager collects data for trauma patients only if they are admitted to hospital for more than 24 hours following an injury.

Demographic, social and clinical information, including age, sex, mode of arrival to hospital, mechanism and place of injury, injury severity scores, length of stay in PICU including days intubated and discharge destination, was extracted from the registry database. The only clinical care variables recorded in the trauma registry were whether or not an operation was performed and if mechanical ventilation was required. Age was categorized into three groups (0–4 years/5–9 years/10–15 years) in accordance with Australian Bureau of Statistics age groupings. Injury severity scores were calculated using the injury severity score (ISS) [20], an anatomical scoring system that provides an overall score for patients with multiple injuries. In the past, major trauma in children was defined with an ISS > 15; however, the recently updated (2017) and nationally recognized Victoria trauma system guidelines define major trauma as serious injury to two or more body systems and an ISS score > 12 [21].

### Data analysis

Summary statistics are presented as median (25^th^-75^th^ percentile) for continuous data and as frequency (percentage) for categorical data. The associations between patient characteristics and mortality for trauma cases recorded since January 2012 were investigated using binary logistic regression models. First, univariable models were constructed, and then multivariable models. In all multivariable models age, year of trauma, and ISS were included as covariables. The results of multivariable models are only presented when there are at least five deaths in each category, in order to avoid overfitting of the model. Effect estimates are presented as odds ratios (OR) and 95% confidence intervals (95%CI). Data analysis was undertaken using Stata statistical software v13.0 (StataCorp, College Station, TX, USA).

## Results

### Demographic data

**Fig 1:**
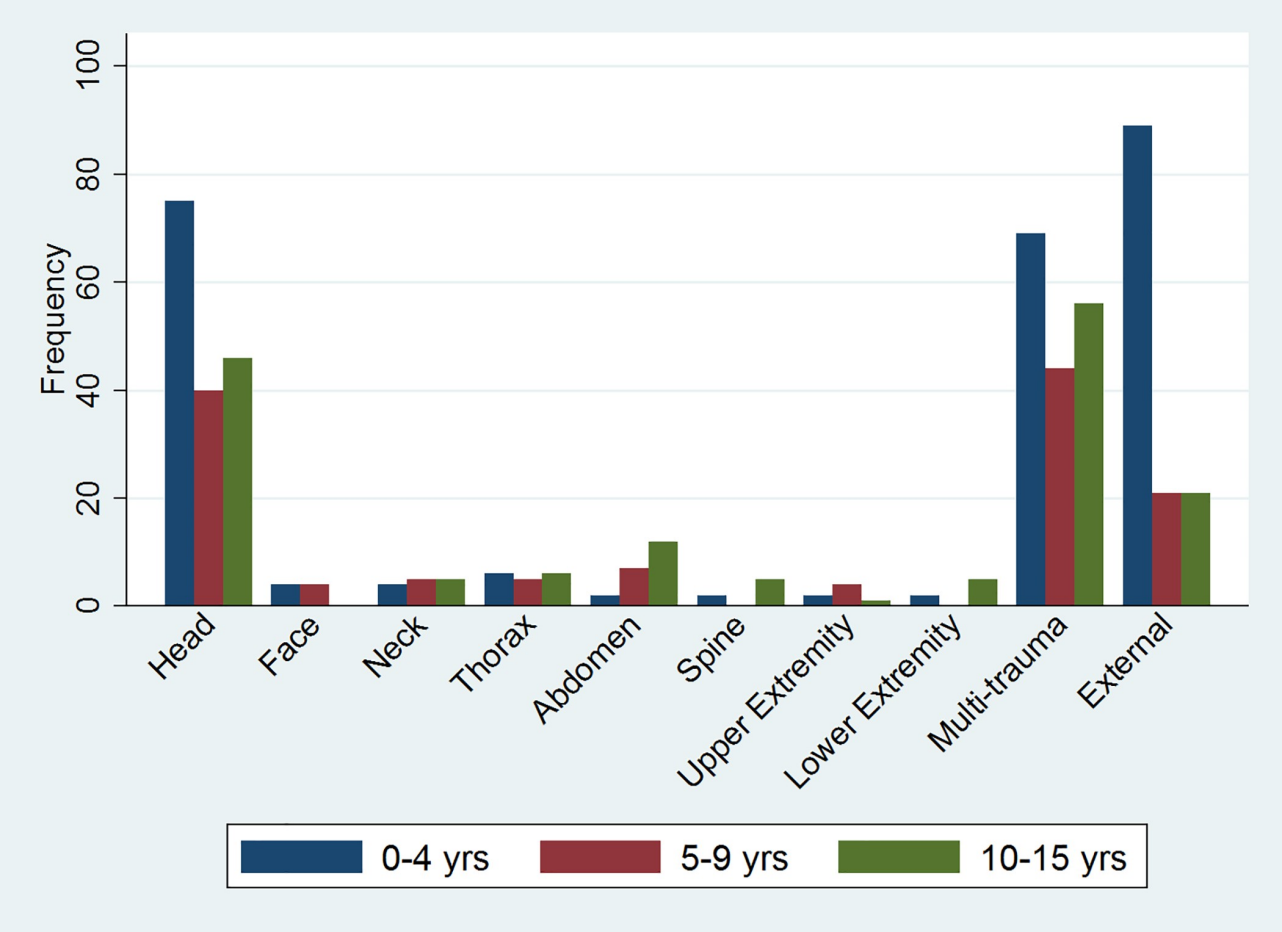
Trauma by body region in demographic groups‡. ‡ (external = burns, drowning, asphyxia and electrical injuries).

There were 542 children admitted to the PICUs in Brisbane following trauma in Queensland during the seven-year study period, and 360 (66.4%) were male. Geographical place of occurrence of the injury was not recorded in two-thirds (n = 357) of the cases, but in 68% (n = 243) of cases in which data were available, the trauma was sustained outside the Brisbane metropolitan area. There were 255 (47.0%) patients aged 0–4 years, 130 (24.0%) aged 5–9 years and 157 (29.0%) aged 10–15 years. The median ISS was 11 (IQR = 9–22), and 261 (48.2%) had ISS > 12 (current definition of major trauma) and 226 (41.7%) had ISS > 15 (previous definition of major trauma). Fig 1 presents the injury patterns across the age groups. Isolated head injury and multi-trauma were the most commonly injured body regions, accounting for nearly equal proportions (approximately 33%) of the total injuries reported in each age group. Of the 47 burns and 54 drowning incidents, 22 (46.8%) and 40 (74.1%) respectively occurred in the 0–4 age group.

### Injury patterns

S1 Table presents demographic and injury event profiles and management by age group. It is notable that all suspected inflicted injury and 93.8% of ingestions (excluding drug overdoses) were seen in the vulnerable 0–4 age group. The home was the most common place of injury among those aged 0–4 years. Hanging was reported only in the 10–15 age group. The roadways and the home environment accounted for a similar proportion of PICU admissions in the 5–9 and 10–15 age groups. The most common mode of arrival at hospital was by ambulance (> 57% in all age groups). The proportion of children who underwent an operative procedure (28.4%) and were intubated/ventilated (37.6%) was similar across all age groups. The median time to triage was 4.8 hours (IQR = 1.5–9.2), and the median length of stay in PICU was 2 days (IQR = 1–4).

### Mortality

Of the 387 admissions post January 2012, 43 (11.1%) patients did not survive to discharge. Patient characteristics associated with mortality are presented in S2 Table. Mortality was not associated with sex. Patients with more severe injuries were less likely to survive. In particular, compared with those with ISS scores of 0–12, those with ISS scores of 18–25 and 26+ had adjusted odds ratios for mortality of 12.9 [95%CI 4.6, 35.8] and 11.7 [95%CI 3.8, 35.8] respectively. Compared with children hospitalized following motor vehicle accidents, the mechanisms of injury with the greatest mortality were inflicted injury (OR = 14.8 [95%CI 2.7, 80.1]), drowning (OR = 30.9 [95%CI 6.7, 143.2]) and hanging (OR = 197.5 [95%CI 15.2, 2567.6]).

## Discussion

This study provides a description of trauma cases admitted to the pediatric intensive care units in Brisbane, Queensland, Australia, between 2008 and 2015. The provision of pediatric severe trauma care within integrated trauma systems can be further improved by ongoing research to guide best practice [22]. Collection of this data has the potential to inform critical care service resource management in Queensland and potentially improve patient outcomes. The data identifies high-risk injury patterns, profiles, severity, management and mortality in different age groups in pediatric major trauma. Higher mortality is associated with abdomen/spine/thorax injuries and with drowning and hanging as the mechanisms of inflicted injury. We identified a vulnerable young population in the 0–4 age group who had the greatest odds of mortality and represented a significant proportion of PICU admissions, with 28% of the overall cohort requiring an operative procedure. Unfortunately, the geographical location of the injury was not recorded in about two-thirds of the patients, so we cannot draw any conclusions about mortality outside the metropolitan area. This shortcoming is being addressed so that the trauma system across the large geographical state of Queensland can be analyzed and improved.

The Children’s Health Queensland Trauma Service Registry captures data prospectively and employs a dedicated, trained data manager to collect, organize and clean data. We are confident that there is minimal missed data and misclassification. It is possible that some patients did not survive their initial injuries out of hospital, and we acknowledge that, by definition, our review does not include this important group of children. Additionally, cases that were admitted for less than 24 hours were not recorded in the registry. Some children aged > 15 years may have been managed in an adult trauma centre. We have identified a number of weaknesses in our data collection whilst undertaking this study and our processes have been improved.

There are several similar studies of pediatric trauma requiring intensive care unit admission. A study by Franzen et al. [17] in Sweden studying pediatric patients (excluding head injury cases which were admitted to a neurosurgical unit) reported a median ISS of 14 with a mean length PICU of stay of 4.2 days and a mortality of 3%. Deasy et al. [15], in Victoria, Australia, reported a median ISS of 25 with a median length of stay of 3 (2–8) days and a mortality of 10%, similar to our overall mortality since January 2012 of 11.1%. The Australian Institute of Health and Welfare estimated that between 2002 and 2004, injury was responsible for 6% of all deaths and approximately 18% and 26% of excess deaths in regional and remote areas respectively [8]. Just over half of our patients (54%) arrived by ambulance, consistent with the findings of the UK Trauma Audit and Research Network report [23]. The home environment was the most common place of injury across all age groups [24, 25]. This is especially notable in the pre-school group who would spend a significant proportion of their time at home. A number of the times to triage were long, with an upper quartile triage time of median time to triage of 9.2 hours, reflecting that size of Queensland and the vast distances some children have to cover before admission.

There were 24 cases of suspected inflicted injury, and 75 cases of isolated head injury in the 0–4 year cohort. The intentional nature of traumatic brain injury has the potential to be missed, and emergency physicians need to have a high index of suspicion; even though the initial clinical findings can be subtle, the subsequent morbidity and mortality are significant [26, 27]. Davies et al. examined the UK Trauma Audit and Research Network data and reported that nearly all cases of severely injured children (97.7%) suffering trauma because of suspected inflicted injury occurred in the 0–5 age group, with 76.3% occurring in infants under the age of 1 year. These patients also had a threefold higher mortality rate [28, 29]. However, a recent New Zealand study found that mortality in inflicted head trauma was highest in children aged > 2 years compared with children < 2 years (38% vs 11%) [30].

The study identified that, based on the injury severity score, almost half (41.7%) of the trauma patients admitted to PICU are severely injured. Caring for these seriously injured patients has an impact on individual personal resources: levels of compassion, energy reserves and the ability to care and feel empathy. Nursing staff are particularly vulnerable as they care for critically injured patients at the bedside for eight- or twelve-hour shifts, sometimes knowing that their efforts are ultimately futile and their patient will die. Although the impact on children admitted to PICUs and their families has been studied [31, 32], there has been minimal research looking at the impact on staff, including compassion fatigue and methods to improve their resilience by using appropriate resources [33], and this is an area where more research would be beneficial.

Our study found that patients aged < 9 years had the highest rates of ventilation. For all children, 57% stayed in the PICU for 1–7 days. The small number of penetrating trauma injuries recorded in the registry is reflective of national trends [34]. Although penetrating trauma is rare in Australia, it may result in serious injury, and the lack of clinical exposure affects the acquisition of surgical skills, expertise and institutional preparedness. In future, linking the trauma and rehabilitation databases could provide functional outcome measures to assess the effects of major trauma on children and their families. One limitation of the database was the absence of recorded physiological parameters at the site of the trauma and the transfer time.

One potential limitation of this study is that the merger of the two paediatric services in 2014 may have altered the trauma system; however, we feel this is unlikely due to the crossover of specialists between the two hospitals, and the joint directorship of the emergency departments of the two hospitals preceding the merger. Further, we are describing the clinical and demographic features of children presenting, which are very unlikely to be influenced by the hospital attended. Likewise, the only outcome we consider is mortality from 2012, a period when both services were under joint directorship with very similar processes for children admitted to PICU.

## Conclusion

This study reports an overall mortality (since January 2012) of 11.1% for severe pediatric trauma with 28% overall requiring an operative procedure. Our study identifies a vulnerable young population in the 0–4 age group who have the greatest odds of mortality and represent a significant proportion of the children admitted to pediatric intensive care units following trauma

## Supporting information

S1 Table Profile of injury patterns and management across age groups. (DOC)

S2 Table Demographic, social and clinical characteristics associated with mortality. Missing items: ISS, n = 1; place of injury, n = 74; mode of arrival, n = 12.
All multivariable models adjusted for age, year of trauma, and Injury Severity Score.
n/c–multivariable logistic regression model not conducted due to small number of events (n<5) in one of the categories.
(DOC)

## Abbreviations

ABS: Australian Bureau of Statistics
AIHW: Australian Institute of Health and Welfare
GCS: Glasgow coma score
ISS: injury severity score
OR: odds ratio
PICU: pediatric intensive care unit
QAS: Queensland Ambulance Service
QTS: Queensland Trauma Service
