# Supplementary material for: A review of children with severe trauma admitted to pediatric intensive care in Queensland, Australia
Source: PLoS One. 2019 Feb 7;14(2):e0211530. doi: 10.1371/journal.pone.0211530 (PMC6366734; doi:10.1371/journal.pone.0211530)
Supplement: S1 Table — (DOC) [file pone.0211530.s001.doc]

***Table 1. Profile of injury patterns and management across age groups 2008-2015 (n=542)***

| **Variable** | | **Overall**  **(n=542)** | **0–4 year**  **(n=255)** | **5–9 years**  **(n=130)** | **10–15 years**  **(n=157)** |
| --- | --- | --- | --- | --- | --- |
| **Sex**, n(%) | Male | 360 (66.4%) | 162 (63.5%) | 80 (61.5%) | 118 (75.2%) |
| **Mechanism, n(%)** | Animal | 30 (5.5%) | 16 (6.3%) | 6 (4.6%) | 8 (5.1%) |
|  | Burns | 47 (8.7%) | 22 (8.6%) | 14 (10.8%) | 11 (7.0%) |
|  | Drowning | 54 (10.0%) | 40 (15.7%) | 7 (5.4%) | 7 (4.5%) |
|  | Hanging | 8 (1.5%) | 0 (0.0%) | 0 (0.0%) | 8 (5.1%) |
|  | High fall (>1m) | 56 (10.3%) | 20 (7.8%) | 20 (15.4%) | 16 (10.2%) |
|  | Low fall (<1 m/ unspecified) | 65 (12.0%) | 25 (9.8%) | 15 (11.5%) | 25 (15.9%) |
|  | Ingestion | 16 (3.0%) | 15 (5.9%) | 1 (0.8%) | 0 (0.0%) |
|  | Inflicted injury | 24 (4.4%) | 24 (9.4%) | 0 (0.0%) | 0 (0.0%) |
|  | Motor vehicle accident | 71 (13.1%) | 35 (13.7%) | 18 (13.8%) | 18 (11.5%) |
|  | Motorbike | 38 (7.0%) | 4 (1.6%) | 9 (6.9%) | 25 (15.9%) |
|  | Pedal cyclist | 6 (1.1%) | 0 (0.0%) | 6 (4.6%) | 6 (3.8%) |
|  | Pedestrian | 25 (4.6%) | 6 (2.4%) | 10 (7.7%) | 9 (5.7%) |
|  | Struck by/collision with person | 14 (2.6%) | 3 (1.2%) | 3 (2.3%) | 8 (5.1%) |
|  | Struck by/collision with object | 48 (8.9%) | 20 (7.8%) | 18 (13.8%) | 10 (6.4%) |
|  | Other mechanism | 40 (7.4%) | 28 (9.8%) | 3 (2.3%) | 6 (3.8%) |
| **Place of injury**, n(%) | Home | 204 (37.6%) | 131 (51.4%) | 37 (28.5%) | 36 (22.9%) |
|  | School | 17 (3.1%) | 2 (0.8%) | 5 (3.8%) | 10 (6.4%) |
|  | Road/street/highway | 101 (18.6%) | 29 (11.4%) | 36 (27.7%) | 36 (22.9%) |
|  | Place of recreation | 46 (8.5%) | 14 (5.5%) | 15 (11.5%) | 17 (10.8%) |
|  | Farm | 16 (3.0%) | 5 (2.0%) | 1 (0.8%) | 10 (6.4%) |
|  | Other | 11 (2.0%) | 4 (1.6%) | 2 (1.5%) | 5 (3.2%) |
|  | Not stated | 147 (27.1%) | 70 (27.5%) | 34 (26.2%) | 43 (27.4%) |
| **Injury severity score**, median(25^th^-75^th^ %ile)  Injury severity score, n(%) |  | 11 (9-22) | 11 (9-25) | 10 (5-19) | 13 (9-22) |
|  | ISS>15  ISS>12 | 226 (41.7%)  261 (48.2%) | 106 (41.6%)  125 (49.0%) | 46 (35.4%)  50 (38.5%) | 74 (47.1%)  86 (54.8%) |
| **Mode of arrival**  **at hospital n(%)** | Ambulance | 318 (58.7%) | 146 (57.3%) | 79 (60.8%) | 93 (59.2%) |
|  | Retrieval/flight | 184 (33.9%) | 87 (34.1%) | 38 (29.2%) | 59 (37.6%) |
|  | Car | 19 (3.5%) | 13 (5.1%) | 4 (3.1%) | 2 (1.2%) |
|  | Missing data | 21 (3.9%) | 9 (3.5%) | 9 (6.9%) | 3 (1.9%) |
| **Body**  **region(AIS),**  **and**  **multi-trauma, n(%)** | Head | 161 (29.7%) | 75 (29.4%) | 40 (30.8%) | 46 (29.3%) |
|  | Face | 8 (1.5%) | 4 (1.6%) | 4 (3.1%) | 0 (0.0%) |
|  | Neck | 14 (2.6%) | 4 (1.6%) | 5 (3.8%) | 5 (3.2%) |
|  | Thorax | 17 (3.1%) | 6 (2.4%) | 5 (3.8%) | 6 (3.8%) |
|  | Abdomen | 21 (3.9%) | 2 (0.8%) | 7 (5.4%) | 12 (7.6%) |
|  | Spine | 7 (1.3%) | 2 (0.8%) | 0 (0.0%) | 5 (3.2%) |
|  | Upper extremity | 7 (1.3%) | 2 (0.8%) | 4 (3.1%) | 1 (0.6%) |
|  | Lower extremity | 7 (1.3%) | 2 (0.8%) | 0 (0.0%) | 5 (3.2%) |
|  | Multi-trauma | 169 (31.2%) | 69 (27.1%) | 44 (33.8%) | 56 (35.7%) |
|  | External and other | 131 (24.2%) | 89 (34.9%) | 21 (16.2%) | 21 (13.4%) |
| **Length PICU stay (days); median (25^th^-75^th^ %ile)** |  | 2 (1-4) | 2 (1-5) | 2 (1-3) | 1 (1-3) |
| **Length PICU stay n (%)** | <1 day | 201 (37.1%) | 96 (37.6%) | 49 (37.7%) | 56 (35.7%) |
|  | 1-3 days  4-7 days | 245 (45.2%)  59 (10.9%) | 105 (41.2%)  33 (12.9%) | 64 (49.2%)  11 (8.5%) | 76 (48.4%)  15 (9.6%) |
|  | 8-14 days | 22 (4.1%) | 14 (5.5%) | 2 (1.5%) | 6 (3.8%) |
|  | 15-150 days | 15 (2.8%) | 7 (2.7%) | 4 (3.1%) | 4 (2.5%) |
| **Time to triage (h); median (25^th^-75^th^ %ile)** |  | 5 (2-9) | 6 (2-10) | 5 (1-9) | 3 (2-9) |
| Time to triage | <1 h | 36 (12.8%) | 17 (14.8%) | 9 (13.4%) | 10 (10.2%) |
|  | 1-3 h | 85 (30.1%) | 25 (21.7%) | 22 (32.8%) | 38 (38.8%) |
|  | 3-6 h | 45 (16.0%) | 21 (18.3%) | 8 (11.9%) | 16 (16.3%) |
|  | 6-12 h | 61 (21.6%) | 28 (24.3%) | 16 (23.9%) | 17 (17.3%) |
|  | 12-24 h | 30 (10.6%) | 14 (12.2%) | 6 (9.0%) | 10 (10.2%) |
|  | >24 h | 23 (8.2%) | 10 (8.7%) | 6 (9.0%) | 7 (7.1%) |
| **Intubated, n(%)** |  | 132 (37.6%) | 62 (38.3%) | 29 (38.7%) | 41 (36.0%) |
| **Operation, n(%)** |  | 154 (28.4%) | 62 (24.3%) | 42 (32.3%) | 50 (31.9%) |
| **Discharge destination, n(%)** | Home | 447 (82.5%) | 200 (78.4%) | 119 (91.5%) | 128 (81.5%) |
|  | Hospital | 13 (2.4%) | 10 (3.9%) | 0 (0.0%) | 3 (1.9%) |
|  | In hospital mortality | 48 (8.9%) | 31 (12.2%) | 6 (4.6%) | 11 (7.0%) |
|  | Not stated | 34 (6.3%) | 14 (5.5%) | 5 (3.8%) | 15 (9.6%) |

Abbreviations: PICU Pediatric Intensive Care Unit; AIS Abbreviated Injury Scale

‘Other mechanism’ includes foreign body aspiration, suspected inflicted/non-accidental injury, gunshot wound, post-delivery trauma, capsized boat, penile trauma, injury with underlying suspected genetic causes, out of hospital cardiac arrest

Time to triage data collected since January 2012.
