# Supplementary material for: A review of children with severe trauma admitted to pediatric intensive care in Queensland, Australia
Source: PLoS One. 2019 Feb 7;14(2):e0211530. doi: 10.1371/journal.pone.0211530 (PMC6366734; doi:10.1371/journal.pone.0211530)
Supplement: S2 Table — Missing items: ISS, n = 1; place of injury, n = 74; mode of arrival, n = 12. All multivariable models adjusted for age, year of trauma, and Injury Severity Score. n/c–multivariable logistic regression model not conducted due to small number of events (n<5) in one of the categories. (DOC) [file pone.0211530.s002.doc]

***Table 2. Demographic, social and clinical characteristics associated with mortality 2012-2015 (n=387)***

| **Covariate** | **Survivors**  **(n = 344)** | **Non-survivors**  **(n = 43)** | **Crude odds ratio**  **(95% CI)** | **Adjusted odds ratio (95% CI)** |
| --- | --- | --- | --- | --- |
| **Age group**, n (%)  0–4 years (reference)  5– 9 years  10–15 years | 149 (84.7%)  84 (94.4%)  111 (91.0%) | 27 (15.3%)  5 (5.6%)  11 (9.0%) | 1.00 (ref.)  0.33 (0.12,0.88)  0.55 (0.26,1.15) | 1.00 (ref.)  0.30 (0.10,0.87)  0.56 (0.25, 1.26) |
| **Sex n (%)**  Male (reference)  Female | 233 (88.9%)  111 (88.8%) | 29 (11.1%)  14 (11.2%) | 1.00 (ref.)  1.01 (0.52, 1.99) | 1.00 (ref.)  0.75 (0.35, 1.59) |
| **Year n (%)**  2012 (reference)  2013  2014  2015 | 84 (89.4%)  100 (88.5%)  86 (83.5%)  74 (84.1%) | 10 (10.6%)  13 (11.5%)  6 (6.5%)  14 (15.9%) | 1.00 (ref.)  1.09 (0.46, 2.61)  0.59 (0.20, 1.68)  1.59 (0.67, 3.79) | 1.00 (ref.)  1.10 (0.43, 2.83)  0.66 (0.21, 2.06)  1.50 (0.58, 3.86) |
| **Injury Severity Score**  ISS 0 – 12  ISS 13 – 15  ISS 16 – 20  ISS 21 – 25  ISS > 26 | 170 (97.1%)  28 (96.6%)  49 (98.0%)  56 (70.0%)  40 (76.9%) | 5 (2.9%)  1 (3.5%)  1 (2.0%)  24 (30.0%)  12 (23.1%) | 1.00 (ref.)  1.21 (0.14, 10.78)  0.69 (0.08, 6.08)  14.6 (5.31, 40.00)  10.2 (3.40, 30.60) | 1.00 (ref.)  n/c  n/c  12.88 (4.63, 35.83)  11.67 (3.81, 35.80) |
| **Body region, burns and multi-trauma n (%)**  Head/face/neck  Abdomen/spine/thorax  Extremity  Multi-Trauma  External and other | 114 (94.2%)  31 (81.6%)  12 (100.0%)  127 (95.5%)  60 (72.3%) | 7 (5.8%)  7 (18.4%)  0 (0.0%)  6 (4.5%)  23 (27.7%) | 1.00 (ref.)  3.67 (1.20, 11.27)  n/c  0.77 (0.25, 2.36)  6.24 (2.53, 15.38) | 1.00 (ref.)  2.95 (0.77, 11.24)  n/c  0.23 (0.06, 0.81)  5.28 (1.76, 15.82) |
| **Injury mechanism n (%)**  Motor vehicle/motorbike accident  Animal  Burns  Inflicted injury  Drowning  Hanging  Fall  Ingestion  Pedal cyclist/pedestrian  Struck by collision with person/object  Other Mechanism | 79 (97.5%)  18 (94.7%)  23 (100.0%)  16 (72.7%)  23 (56.1%)  1 (16.7%)  82 (98.8%)  8 (88.9%)  29 (100.0%)  42 (97.7%)  23 (74.2%) | 2 (2.5%)  1 (5.3%)  0 (0.0%)  6 (27.3%)  18 (43.9%)  5 (83.3%)  1 (1.2%)  1 (11.1%)  0 (0.0%)  1 (2.3%)  8 (25.8%) | 1.00 (ref)  2.19 (0.19, 25.54)  n/c  14.81 (2.74, 80.12)  30.91 (6.67, 143.18)  197.50(15.19,2567.56)  0.48 (0.04, 5.42)  4.94 (0.40, 60.65)  n/c  0.94 (0.08, 10.68)  13.74 (2.73, 69.26) | n/c  n/c  n/c  n/c  n/c  n/c  n/c  n/c  n/c  n/c  n/c |
| **Place of injury n (%)**  Home  School  Road/street/highway  Place of recreation  Farm  Other | 132 (83.0%)  8 (88.9%)  84 (95.5%)  32 (86.5%)  13 (92.9%)  5 (83.3%) | 27 (17.0%)  1 (11.1%)  4 (4.6%)  5 (13.5%)  1 (7.1%)  1 (16.7%) | 1.00 (ref.)  0.61 (0.08, 6.20)  0.27 (0.09, 0.83)  0.95 (0.32, 2.85)  0.62 (0.07, 5.35)  0.90 (0.09, 8.49) | 1.00 (ref.)  n/c  n/c  0.76 (0.23, 2.54)  n/c  n/c |
| **Mode of arrival to hospital n (%)**  Ambulance  Retrieval/flight  Car | 167 (87.0%)  151 (91.0%)  17 (100.0%) | 25 (13.0%)  15 (9.0%)  0 (0.0%) | 1.00 (ref.)  0.67 (0.34, 1.31)  n/c | 1.00 (ref.)  0.56 (0.26, 1.18)  n/c |

Missing items: ISS, n=1; place of injury, n=74; mode of arrival, n=12.

All multivariable models adjusted for age, year of trauma, and Injury Severity Score.

n/c – multivariable logistic regression model not conducted due to small number of events (n<5) in one of the categories.
